# Fecal filtrate transplantation attenuates liver and gut injury in experimental alcohol-associated liver disease

**DOI:** 10.1128/spectrum.00377-26

**Published:** 2026-09-22

**Authors:** Nishu Choudhary, Ashi Mittal, Kavita Yadav, Jaswinder Singh Maras, Shiv K. Sarin, Shvetank Sharma

**Affiliations:** 1 Department of Molecular and Cellular Medicine, Institute of Liver and Biliary Sciences 80402, New Delhi, India; 2 Department of Hepatology, Institute of Liver and Biliary Sciences 80402, New Delhi, India; Cleveland Clinic Lerner Research Institute, Cleveland, Ohio, USA

**Keywords:** alcohol-associated liver disease, gut microbiota, fecal filtrate transplant, metabolome

## Abstract

Fecal microbiota transplantation (FMT) carries a risk of infection. Cell-free fecal filtrate transplantation (FFT) represents a potentially safer alternative. The benefit of FFT has not been explored in alcohol-associated liver disease (ALD). We evaluated the therapeutic potential of FFT in a murine model of ALD. C57BL/6N mice were pair-fed control or ethanol Lieber-DeCarli diets with thioacetamide for 12 weeks to induce ALD. FFT (0.22-µm-filtered stool slurry) from healthy mice was administered three times per week to ALD animals. Post-FFT day 7, hepatic and intestinal injury and inflammation were assessed. Fecal microbiota was assessed by 16S rRNA sequencing, and the hepatic/stool metabolome was assessed by mass spectrometry. FFT was significantly better than abstinence, with reduced hepatic pro-inflammatory markers at the protein level, including IL-6 (1.6 fold change [FC], *P* < 0.001), TNF-α (1.2 FC, *P* < 0.001), and also decreased collagen deposition (2.97 FC, *P* = 0.003) and TGF-β expression (2 FC, *P* < 0.001). FFT improved intestinal barrier integrity by upregulation of ZO-1 protein (1.5-fold, *P* = 0.002) and Muc2 mRNA (3.28 FC, *P* = 0.007), with concomitant reduction of plasma-endotoxin (2.5 FC, *P* < 0.001). FFT suppressed pathogenic taxa like *Escherichia–Shigella* (log2 FC = −9.5, padj = 3.42E-13), *Proteus* (log2 FC = −5.6, padj = 0.004), *Desulfovibrio* (log2 FC = −1.5, padj = 0.004), and increased *Prevotellaceae* NK3B31 group (log2 FC = 2.7, padj = 0.018), and *Acinetobacter* (log2 FC = 4.9, padj = 9.41E-16) in the gut. Metabolomic profiling of stool revealed significant increases (*P* < 0.05) in butyric, valeric, isobutyric, and ursodeoxycholic acids. These metabolites enriched pathways like butyrate metabolism (*P* = 0.04), mitochondrial β-oxidation (*P* = 0.008), and PPARα signaling (*P* = 0.01), indicating intestinal and hepatic repair. FFT confers hepatoprotection in ALD by attenuating inflammation and fibrosis, and restoring the intestinal barrier, gut microbiota composition, and beneficial SCFA and bile acid metabolism. These findings support FFT as a promising preclinical strategy for gut–liver axis modulation in ALD.

**IMPORTANCE:**

Alcohol-associated liver disease (ALD) involves disrupted gut-liver communication, contributing to inflammation, impaired intestinal barrier function, and metabolic disturbances that exacerbate liver injury. This study demonstrates that fecal filtrate transplantation (FFT) attenuates alcohol-induced liver injury while modulating intestinal barrier-related markers, gut microbial communities, and microbial metabolites. These findings highlight the potential of cell-free microbial components as modulators of the gut–liver axis and provide a basis for developing alternative approaches to gut-based intervention in ALD.

## INTRODUCTION

Among the patients with alcohol-associated liver disease (ALD), a majority develop steatosis, with only 10%–35% developing hepatitis, of whom 8%–20% eventually advance to cirrhosis (1). The pathophysiology of ALD is influenced by compositional shifts in intestinal bacteria (2–4). Alcohol intake reduces bacterial diversity, with an increase in the abundance of *Proteobacteria* and *Enterobacteria* and a decrease in the abundance of *Bacteroidetes* and Firmicutes, specifically *Lactobacillus* species (3–5).

Currently, one of the effective treatments for ALD is restoring gut bacterial communities by fecal microbiota transfer (FMT) (5–8), which has been shown to improve 90-day survival in patients with severe alcoholic hepatitis (9–11). There is a lack of response in a subset of patients, probably due to the condition of the patient or suboptimal donor microbiota composition (12). To establish FMT as an efficient, practical therapy for ALD, strategies are required to ensure improved engraftment of donor microbiota.

During FMT, in addition to the microbiota, stool slurry containing debris, oligonucleotides, and other biochemical exudates is also transferred. However, fecal microbiota transplantation has been shown to induce infection in some patients (13). To address this limitation, a new approach has been recognized, known as a fecal filtrate transplant (FFT), in which stool is sterile-filtered to remove all the small particles and microbial cells, reducing the likelihood of invasive bacterial infections. FFT represents an innovative therapeutic approach in liver disease management, focusing on transferring microbial components without intact bacterial cells, and offers a potentially safer alternative to traditional FMT. It has been shown to be equally beneficial to FMT in treating *Clostridium difficile* infections, alleviating symptoms for at least 6 months (14). FFT is also known as fecal virome transplantation (FVT) since viruses do not get filtered out during this process. Obese mice administered FVT from thin donors exhibited a 15% reduction in weight gain and a complete normalization of blood glucose tolerance compared with obese control mice (15). This effect was potentially mediated by FVT-induced alterations in gut microbiota, a finding consistent with another study involving antibiotic-treated mice (16). In the study, mice receiving viable phages showed microbiota profiles more closely resembling their pre-antibiotic state than those receiving non-viable phages, suggesting FFT could serve as a safe alternative to whole microbiota transplants. Despite these promising findings, FFT has not yet been explored in ALD, warranting further investigation into its potential therapeutic applications.

To address this lacuna, we performed FFT in ALD mice and found that FFT confers hepatoprotection in ALD by simultaneously reducing inflammation and fibrosis, restoring intestinal barrier function, reshaping gut microbiota, and enhancing beneficial SCFA and bile acid metabolism. By avoiding whole-bacterial transfer, FFT represents a safe, mechanistically distinct, and potentially translatable therapeutic strategy for ALD, warranting further investigation.

## RESULTS

### Fecal filtrate transplantation improves liver function and injury in ALD mice

**Fig 1:**
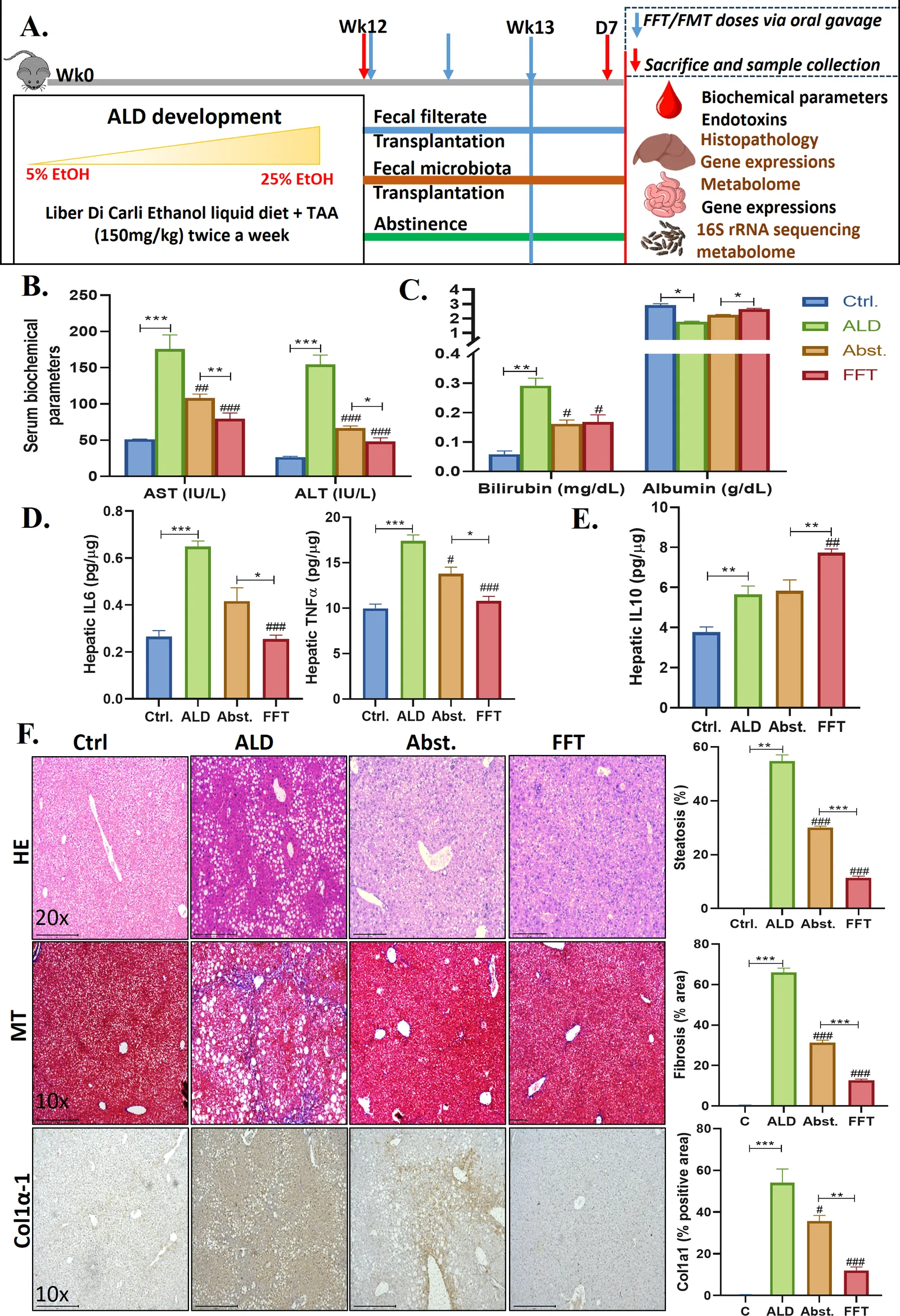
FFT improves liver function and injury in ALD mice. (*n* = 6 mice/group) (**A**) FFT was performed in ALD mice, and mice were sacrificed 7 days post-FFT. Tissues were collected for further assessment. (**B**) Serum AST and ALT; (**C**) bilirubin and albumin levels; (**D**) protein levels of hepatic Il6 and Tnfα; and (**E**) IL-10 (**F**) Representative micrographs of liver histology by hematoxylin and eosin (H&E), Masson’s trichrome (MT) stain, and immunohistochemistry of collagen I (Col1α1). Statistical significance was assessed by one-way ANOVA/Kruskal-Wallis test, as appropriate, with post hoc comparisons. Data are presented as mean ± SEM (standard error mean). *P < 0.05, **P < 0.01, ***P < 0.001 show intergroup statistics; #*P* < 0.05, ##*P* < 0.01, ###*P* < 0.001 are with respect to the ALD group.

Prior to administration, freshly prepared FFT was evaluated by aerobic bacterial culture on Luria-Bertani (LB) agar plates to assess the efficiency of the filtration procedure. No detectable bacterial colony formation was observed after 48 h of incubation, whereas the corresponding unfiltered fecal slurry (positive control) exhibited abundant bacterial growth, supporting effective removal of culturable aerobic bacteria by the 0.22-μm filtration procedure (Fig. S1A). FFT was subsequently administered to ALD mice, with the abstinence group serving as the control (Fig. 1A). Both abstinence and FFT significantly increased body weight in ALD mice (Fig. S1B), with FFT significantly decreasing the liver-to-body-weight ratio (Fig. S1C). Although abstinence improved the liver injury, FFT produced a greater improvement. Compared to abstinence, FFT significantly reduced liver injury markers: AST (1.35, *P* = 0.004), ALT (1.38 FC, *P* = 0.04), and bilirubin (1.04 FC, *P* = 0.04) (Fig. 1B and C). Serum albumin also improved post-FFT (1.18 FC, *P* = 0.003, Fig. 1C). FFT markedly suppressed hepatic pro-inflammatory mediators at both the mRNA (Fig. S1D) and protein levels compared to ALD, including IL-6 (2.6-fold, *P* < 0.001) and TNFα (1.6-fold, *P* < 0.001) (Fig. 1D), while significantly upregulating anti-inflammatory IL-10 (1.3-fold, *P* = 0.004; Fig. 1E). Relative to abstinence, FFT further decreased IL-6 (1.6-fold, *P* = 0.01) and TNFα (1.2-fold, *P* = 0.01), and increased IL-10 (1.3-fold, *P* = 0.009). The mRNA levels of profibrogenic genes were also significantly downregulated in FFT (Acta2 3.1 FC and Tgfβ 2.8 FC, both *P* < 0.001; Fig. S1E). Importantly, when compared directly with abstinence, FFT further decreased fibrogenic gene expression (Acta2 1.8 FC, *P* = 0.04 and Tgfβ 2 FC, *P* < 0.001).

Histopathological analysis confirmed these results (Fig. 1F). FFT markedly reduced hepatic steatosis (4.9-fold) and fibrosis (5.5-fold) compared with ALD mice (*P* < 0.001). Consistent with this, immunohistochemical staining showed that FFT significantly reduced hepatic collagen I (Col1α1) deposition compared with ALD mice (4.5-fold, *P* < 0.001). Relative to abstinence, FFT conferred additional benefits, further decreasing steatosis (2.5-fold), fibrosis (2.7-fold; *P* < 0.001), and Col1α1 (2.97-fold, *P* = 0.003). Thus, FFT offers superior hepatic protection by alleviating steatosis, fibrosis, and inflammation in ALD mice.

### FFT restores intestinal barrier integrity

**Fig 2:**
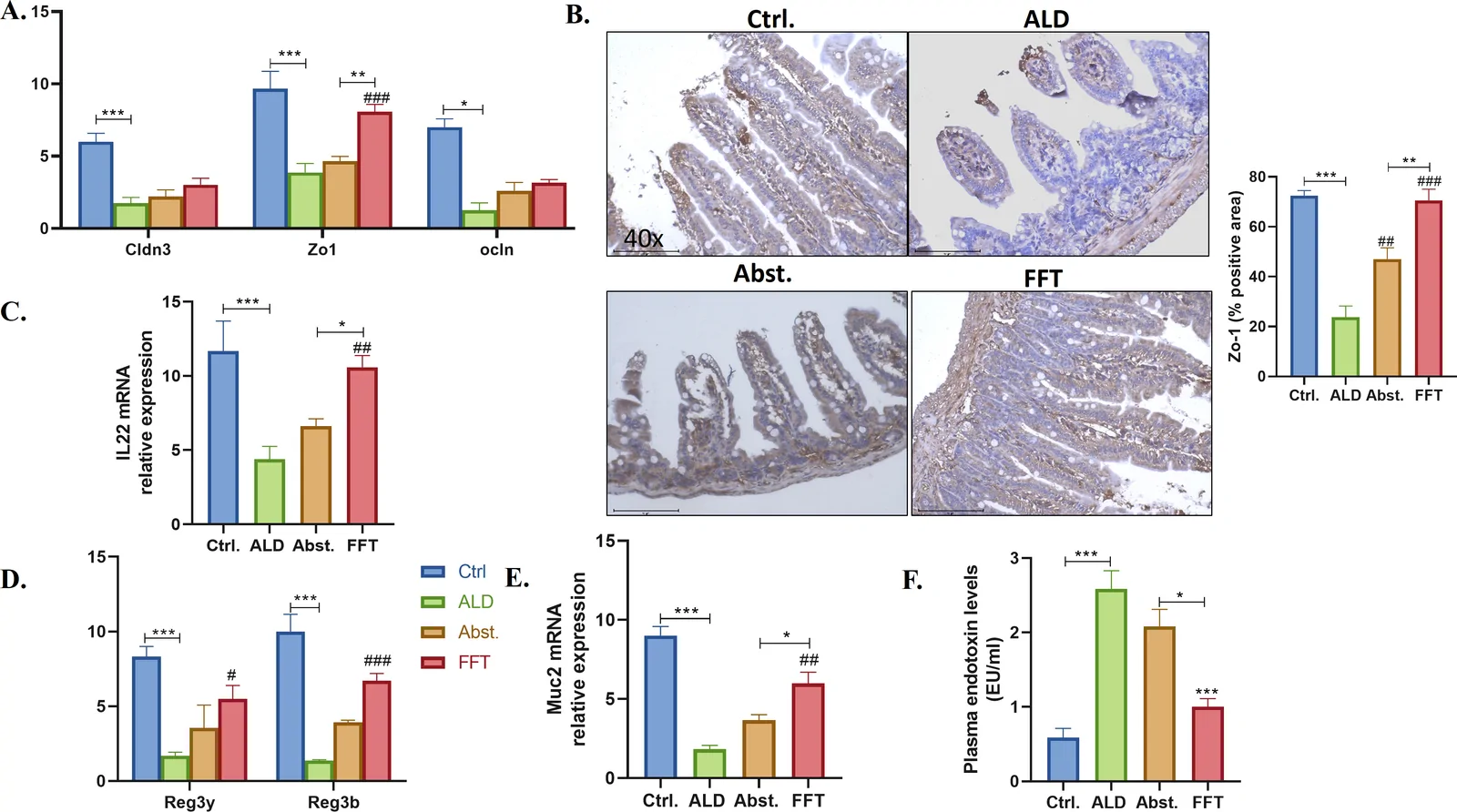
FFT restores intestinal barrier integrity. (*n* = 6 mice/group) (**A**) mRNA expression of ileal tight junction proteins: ZO-1, Cldn3, and Ocln. (**B**) Representative immunohistochemistry and quantification of ZO-1 protein expression. (**C**) mRNA expression of IL-22. (**D**) mRNA expression of antimicrobial peptides Reg3β and Reg3γ. (**E**) Muc2 mRNA expression. (**F**) Plasma endotoxin levels. Statistical significance was assessed by one-way ANOVA/Kruskal-Wallis test, as appropriate, with post hoc comparisons. Data are presented as mean ± SEM (standard error mean). *P < 0.05, **P < 0.01, ***P < 0.001 show intergroup statistics; #*P* < 0.05, ##*P* < 0.01, ###*P* < 0.001 with respect to the ALD group. *Cldn3: claudin 3; Ocln: occludin; Muc2: mucin 2*

FFT significantly upregulated ZO-1 mRNA expression (2.1-fold, *P* = 0.001 vs. ALD; 1.7-fold, *P* = 0.008 vs. abstinence), while no significant changes were observed in claudin-3 or occludin expression (Fig. 2A). Consistent with this, immunohistochemical staining showed a significant increase in ZO-1 protein expression following FFT (2.97-fold, *P* < 0.001 vs. ALD; 1.5-fold, *P* = 0.002 vs. abstinence; Fig. 2B). FFT also significantly increased intestinal IL-22 mRNA expression (2.4-fold, *P* = 0.02 vs. ALD; 1.6-fold, *P* = 0.04 vs. abstinence; Fig. 2C), a cytokine known to drive antimicrobial peptide production in the gut(17). The antimicrobial peptides Reg3β and Reg3γ significantly increased following FFT (5.1 FC, *P* < 0.001, and 3.37 FC, *P* = 0.01, respectively; Fig. 2D). Alcohol consumption degrades Muc2 in the gut mucus lining, contributing to a leaky gut and elevated endotoxemia (18). Compared to ALD, FFT enhanced Muc2 gene expression (3.28 FC, *P* = 0.007; Fig. 2E), with a concomitant reduction in plasma endotoxin levels (2.5 FC, *P* < 0.001, Fig. 2F). Interestingly, when compared with abstinence, FFT increased Muc2 by 1.63 FC, *P* = 0.04, and decreased endotoxin by 2 FC, *P* = 0.01. These findings demonstrate that FFT strengthens intestinal barrier function by enhancing tight junction integrity, increasing IL-22 expression, upregulating antimicrobial peptides, and restoring mucus production, ultimately leading to reduced endotoxemia.

Next, we compared FFT with FMT and found that both significantly alleviated alcohol-induced hepatic injury, though the effects were more pronounced with FMT (Fig. S2). Serum biochemical parameters showed that while FFT reduced AST and ALT levels relative to ALD, FMT led to a further decline in AST (1.33 FC, *P* = 0.01) and ALT (1.5 FC, *P* = 0.06) (Fig. S2A). Similarly, total bilirubin levels were lower in FMT-treated mice (2.2 FC, *P* = 0.04), while albumin levels were higher compared to FFT (Fig. S2B). Histological analysis revealed reduced hepatic steatosis and fibrosis in both groups, with FMT producing a greater reduction in steatosis (1.32 FC, *P* = 0.02; Fig. S2C).

At the intestinal level, both treatments upregulated tight junction genes, though FMT induced stronger effects. Specifically, FMT significantly enhanced Cldn3 and ZO-1 expression (*P* < 0.05 vs FFT), while Ocln remained unchanged between the two groups (Fig. S2D). In parallel, plasma endotoxin levels were markedly reduced following both treatments, with FMT showing a greater decrease (1.69 FC, *P* = 0.03; Fig. S2E).

Overall, these findings indicate that although FMT exhibits superior efficacy in restoring hepatic and intestinal integrity, FFT still confers substantial protection compared to abstinence, suggesting its potential as a safer, bacteria-free therapeutic alternative for managing ALD. FMT was included as a comparator to benchmark FFT’s efficacy, while subsequent mechanistic analyses focused on FFT, since its mechanistic basis in ALD was previously unexplored, unlike FMT (5–7, 9, 10).

### FFT restores microbial homeostasis

**Fig 3:**
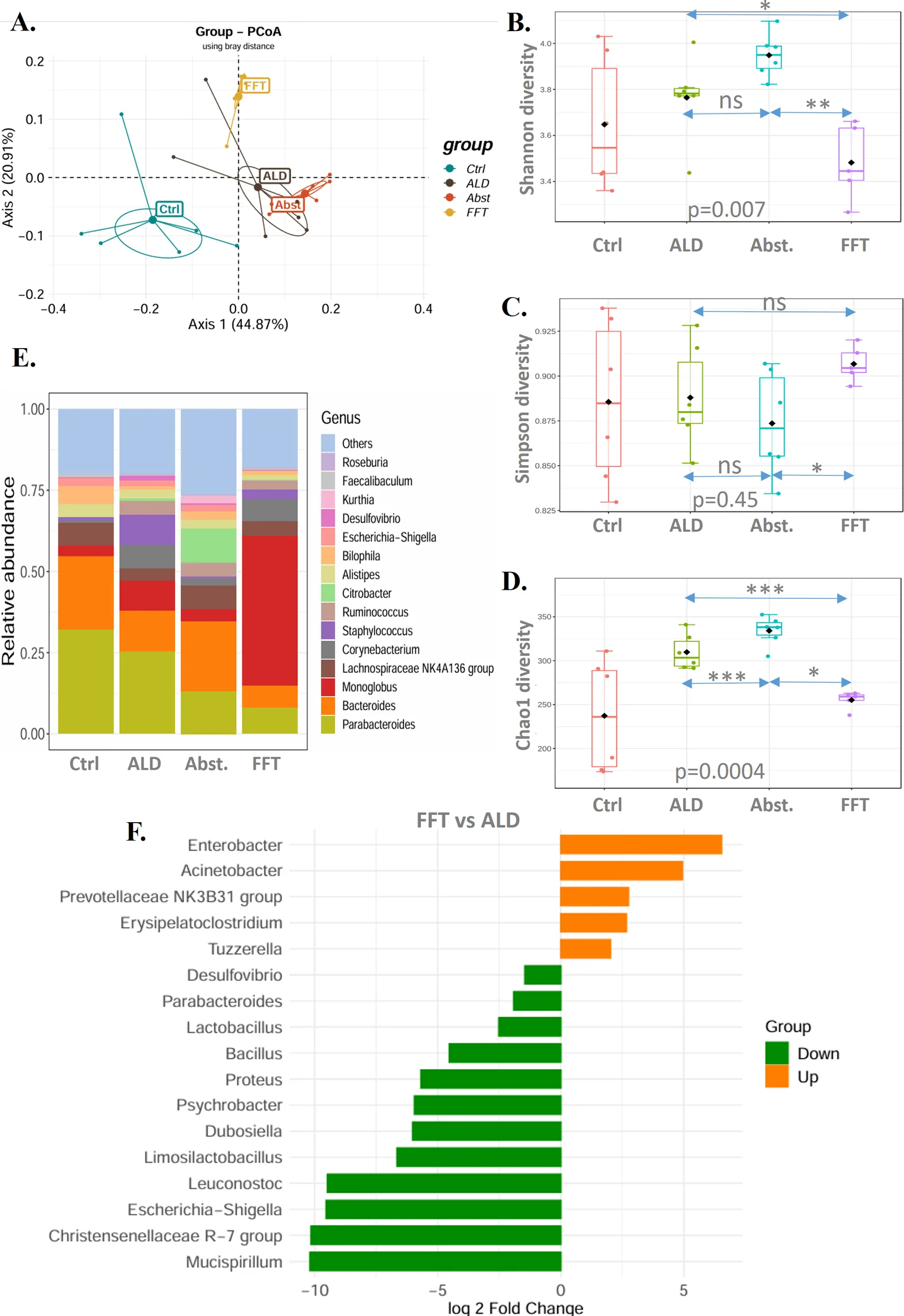
FFT restores microbial homeostasis. (n = 6 mice/group). (**A**) PCoA (calculated using Bray-Curtis distances) plot showing variation in microbial composition. (**B**) Bar plot showing Shannon, (**C**) Simpson, and (**D**) Chao1 diversity (statistical significance was assessed by one-way ANOVA). (**E**) Comparison of relative abundance of bacterial genera (**F**) Histogram showing differentially altered taxa between FFT and ALD (assessed using DESeq2 (SHAMAN pipeline), with Benjamini–Hochberg FDR-adjusted P values). *padj < 0.05, **padj < 0.01, ***padj < 0.001. *PCoA: principal coordinate analysis*.

Gut microbiota composition was markedly altered across groups. Principal coordinate analysis (PCoA) based on Bray-Curtis distance showed distinct clustering of ALD, abstinence, and FFT groups compared to controls, with FFT forming a separate cluster from both ALD and abstinence (*P* = 0.001; Fig. 3A). Microbial diversity analyses revealed a nonsignificant reduction in α-diversity (Shannon and Simpson indices) with alcohol feeding compared to controls (Fig. 3B through D). Abstinence showed the highest α-diversity among all groups, exceeding even controls, whereas FFT did not significantly increase diversity relative to ALD.

At the taxonomic level, relative abundance plots demonstrated that alcohol feeding increased pathogenic genera such as *Escherichia–Shigella* and *Staphylococcus*, while reducing beneficial taxa including *Parabacteroides and Bilophila* (Fig. 3E). FFT treatment reshaped the gut microbiota composition, leading to a relative increase in *Monoglobus* (associated with fiber metabolism), and significant decrease in pathogenic taxa (*Escherichia–Shigella* (log2 FC = −9.5, padj = 3.42E-13), *Proteus* (log2 FC = −5.6, *P* = adj.004), *Desulfovibrio* (log2 FC = −1.5, padj = 0.004)), and enrichment of *Prevotellaceae NK3B31 group* (log2 FC = 2.7, padj = 0.018)*, and Acinetobacter* (log2 FC = 4.9, padj = 9.41E-16); Fig. 3F; Table S2). Together, these findings indicate that while abstinence modestly restores microbial diversity, FFT more effectively remodels gut microbial composition by suppressing pathogenic bacteria and enriching beneficial genera in ALD mice.

### Stool metabolomic profile following FFT treatment in ALD mice

**Fig 4:**
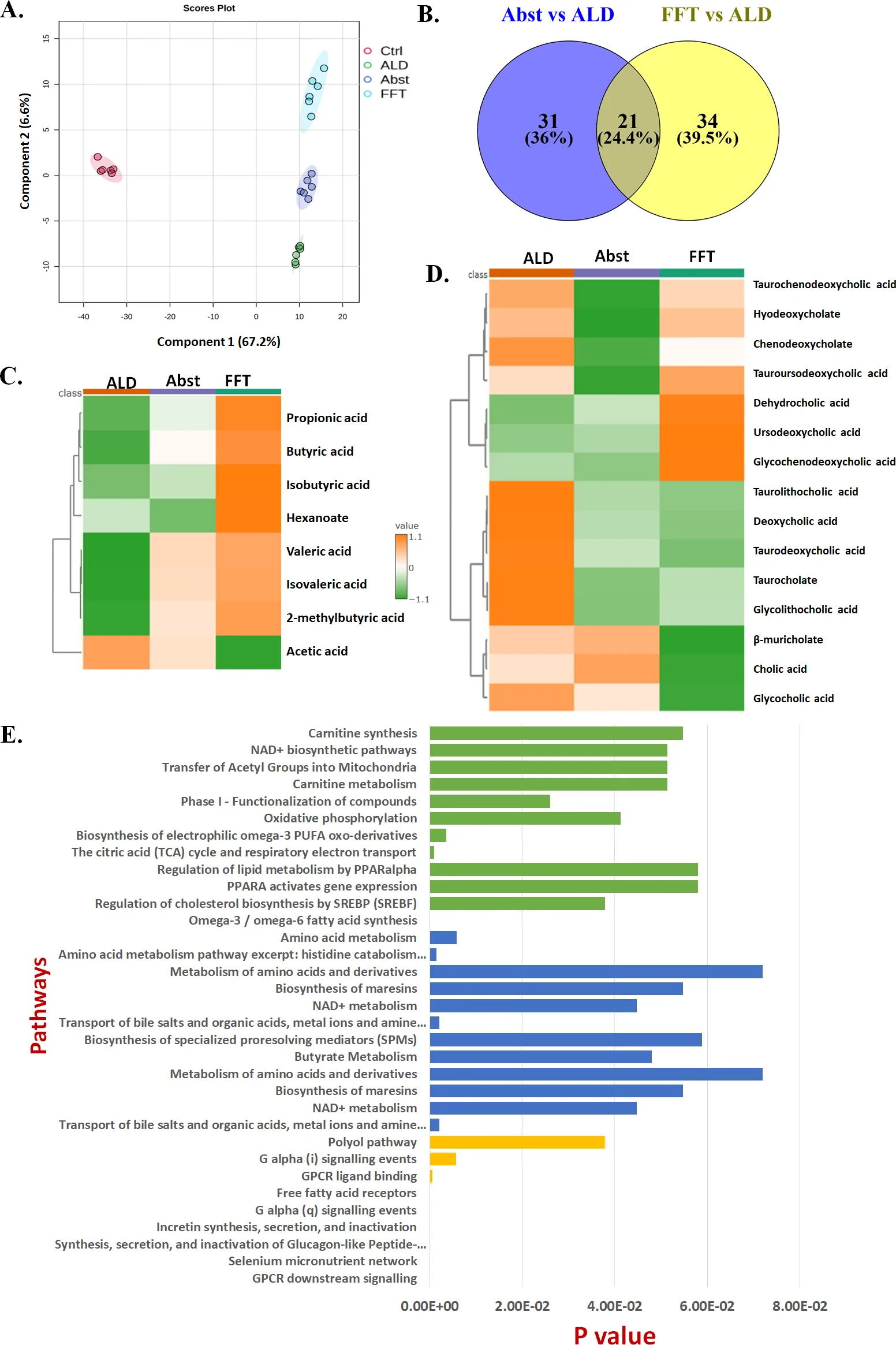
Stool metabolomic profiling in response to fecal filtrate transplantation in ALD. (n = 6 mice/group). (**A**) PLS-DA plot showing approx 74% variance in metabolic composition between the groups. (**B**) Significantly upregulated common and unique DEMs. (**C**) Hierarchical clustering heatmap of bile acids and (**D**) SCFAs showing elevation of ursodeoxycholic acid, tauroursodeoxycholic acid, butyrate, and propionate in the FFT group. (**E**) Pathway enrichment analysis of upregulated metabolites showing upregulated pathways related to intestinal and hepatic recovery. PLS-DA: partial least squares discriminant analysis; DEMs: differentially expressed metabolites; SCFAs: short-chain fatty acids.

FFT alters the stool metabolomic profile, identified 665 annotated metabolites (m/z and RT), and PLS-DA demonstrated distinct clustering of experimental groups, indicating marked differences in metabolite profiles among the treatment groups (Fig. 4A). Model robustness was further evaluated by 5-fold cross-validation, which demonstrated high predictive performance based on classification accuracy, R², and Q². In addition, permutation testing (100 permutations) supported the conclusion that the observed class separation was unlikely to have occurred by chance (Fig. S3A and B). Differential analysis (fold change >1.5, *P* < 0.05) revealed 115 DEMs between the ALD and abstinence groups (51 upregulated, 63 downregulated) and 162 DEMs between the FFT and ALD groups (55 upregulated, 107 downregulated; Fig. S2A and B; Table S3); among these, 34 were uniquely upregulated by FFT, and 21 overlapped with abstinence (Fig. 4B; Table S4). Consistent with previous evidence that SCFAs and bile acids critically mediate gut–liver axis recovery in ALD (19–24), FFT significantly increased stool butyric acid (*P* = 2.31E-05), valeric acid (*P* = 0.01), and isobutyric acid (*P* = 0.008), while reducing acetic acid (*P* = 3.97E-05; Fig. 4D). Ursodeoxycholic acid (*P* = 0.003) and chenodeoxycholic acid (*P* = 0.09) were elevated, whereas deoxycholic acid was reduced (*P* = 0.002; Fig. 4C; Table S5), indicating a shift toward hepatoprotective bile acid species (25–27). Pathway enrichment of upregulated metabolites highlighted intestinal recovery pathways (pathway involves free fatty acid receptors, *P* = 5.91E-05; GPCR ligand binding, *P* = 0.005; selenium micronutrient network, *P* = 2.09E-05; GLP-1 synthesis/secretion/inactivation, *P* = 4.46E-05), hepatic recovery pathways (oxidative phosphorylation, *P* = 0.04; biosynthesis of electrophilic ω−3 PUFA oxo-derivatives, *P* = 0.003; PPARA activation, *P* = 0.05; cholesterol biosynthesis regulation by SREBF, *P* = 0.03), and shared gut–liver axis pathways (butyrate metabolism, *P* = 0.04; biosynthesis of specialized pro-resolving mediators [SPMs], *P* = 0.05; amino acid metabolism and derivatives, *P* = 0.001; transport of bile salts, and organic acids, metal ions, and amine compounds, *P* = 0.002; Fig. 4E; Table S6).

Collectively, these findings demonstrate that FFT reprograms the stool metabolome toward SCFA- and bile acid–driven pathways that support intestinal repair, hepatoprotection, and restoration of the gut–liver axis in ALD.

### Hepatic metabolomic shifts following FFT in ALD mice

**Fig 5:**
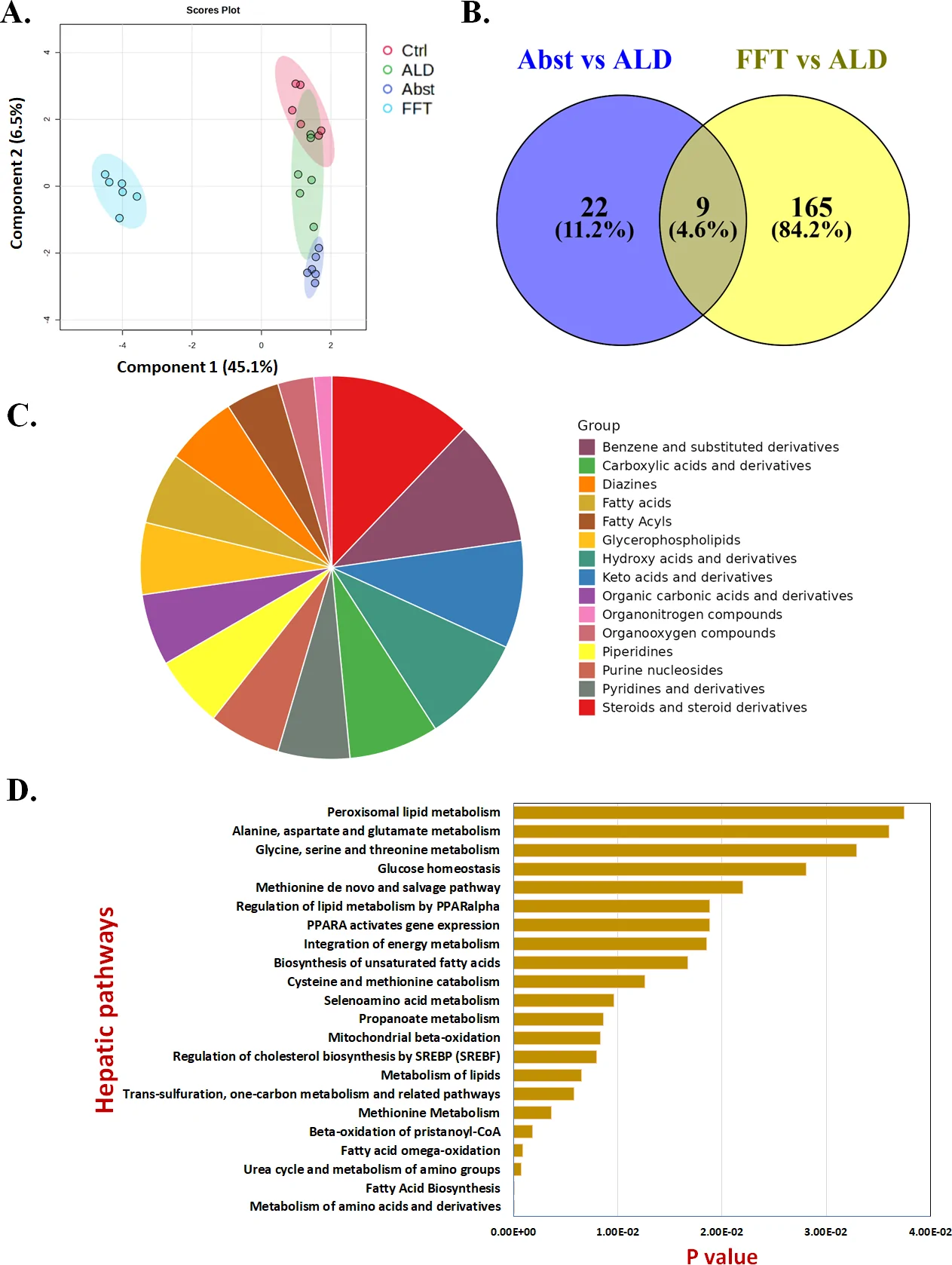
Hepatic metabolomic profiling in response to fecal filtrate transplantation in ALD. (n = 6 mice/group) (**A**) PLS-DA plot showing approx 51% variance in metabolic composition between the groups (**B**) Significantly upregulated common and unique DEMs. (**C**) Pie chart showing metabolic classes of significantly (*P* < 0.05) upregulated metabolites in FFT. (**D**) Pathway enrichment analysis of upregulated metabolites showing upregulated hepatic pathways. *PLS-DA: partial least squares discriminant analysis; DEMs: differentially expressed metabolites*.

To assess the impact of FFT on hepatic metabolism underlying the observed recovery, comprehensive profiling was performed, identifying 400 annotated metabolites (m/z and RT). PLS-DA revealed distinct separation among the experimental groups, indicating substantial metabolic remodeling in the liver following FFT treatment (Fig. 5A). The robustness of the PLS-DA model was confirmed by 5-fold cross-validation and permutation testing (Fig. S4A and B). Differential analysis (FC >1.5, *P* < 0.05) identified 33 DEMs in the abstinence vs ALD comparison (31 upregulated, two downregulated), while the FFT vs ALD comparison revealed 259 DEMs (174 upregulated, 85 downregulated; Fig. S4A and B; Table S7). Consistent with the more pronounced hepatic recovery in FFT animals, 165 metabolites were uniquely upregulated by FFT, with only nine overlapping with abstinence (Fig. 5B; Table S8). These FFT-unique metabolites spanned multiple functional classes, including fatty acids (*P* = 0.03), organonitrogen compounds (*P* = 0.0005), benzene and substituted derivatives (*P* = 0.001), and bile acids (*P* = 0.03; Fig. 5C and Table S9).

Pathway enrichment analysis of these FFT-exclusive metabolites highlighted recovery-associated pathways, including regulation of cholesterol biosynthesis by SREBP (*P* = 0.007), mitochondrial β-oxidation (*P* = 0.008), urea cycle and amino group metabolism (*P* = 0.0007), PPARα activation (*P* = 0.01), methionine metabolism (*P* = 0.003), and alanine, aspartate, and glutamate metabolism (*P* = 0.03; Fig. 5D and Table S10). These pathways align with restoration of mitochondrial function, lipid handling, nitrogen balance, and one-carbon metabolism. Collectively, these data suggest that FFT reshapes the hepatic metabolome toward enhanced mitochondrial energy production, lipid utilization, and amino acid turnover—metabolic rewiring processes known to counteract steatosis and oxidative stress in ALD (28, 29).

### Correlation analysis among liver injury markers, gut microbiota, and stool microbial metabolites

**Fig 6:**
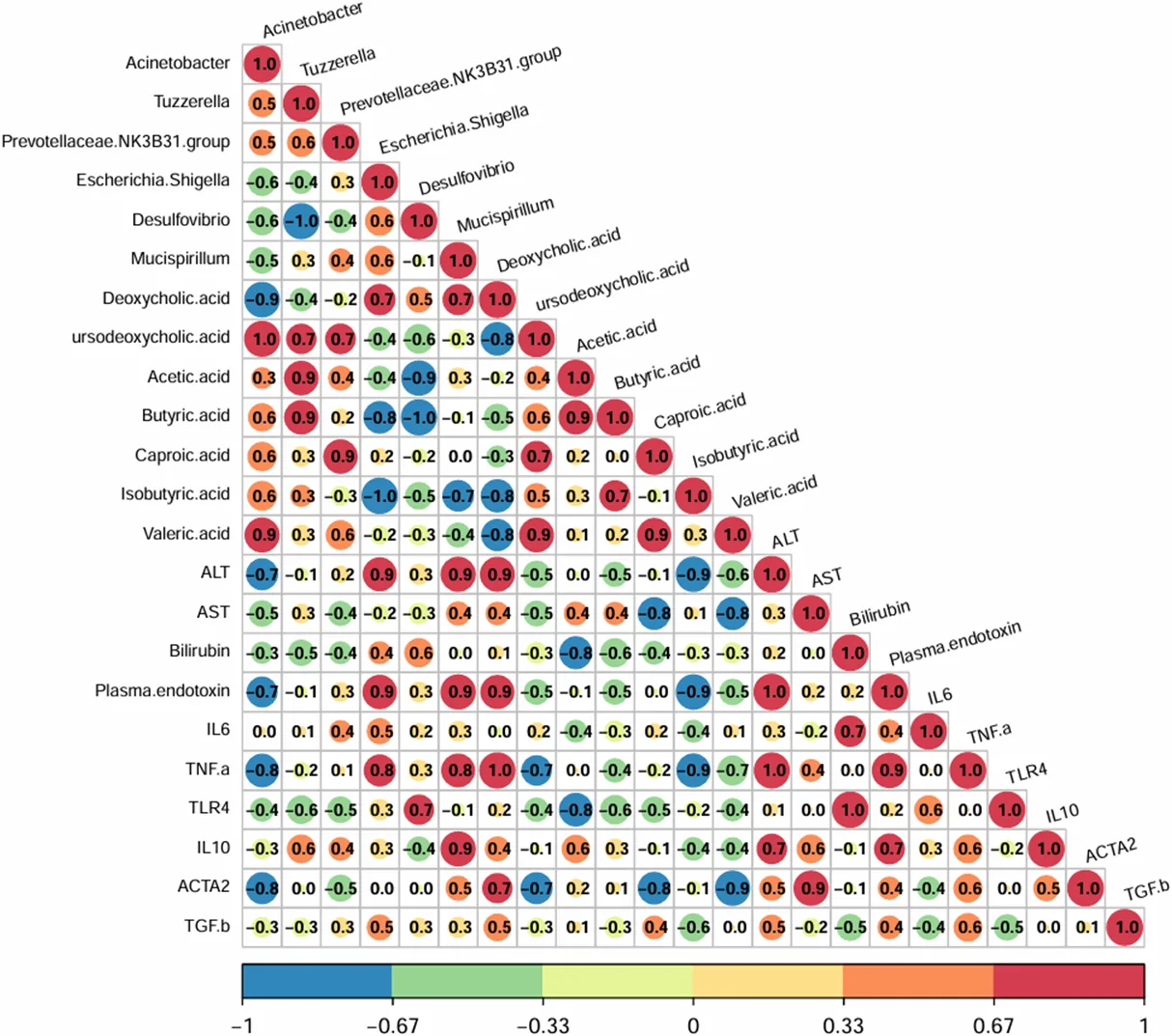
Correlation analysis between liver injury markers, gut microbiota, and microbial metabolites altered by FFT. (*n* = 6 mice/group). Pearson’s correlation values are displayed in circle. Red color shows the positive correlation, while blue color shows the negative correlation. Correlation according to the scale given at the bottom of the plot. (Pearson’s correlation = r² > 0.7, *P* < 0.05)

A correlation analysis of differentially abundant gut bacterial taxa with liver injury biomarkers and microbial metabolites in the FFT (r² > 0.7, *P* < 0.05; Fig. 6; Table S11) revealed distinct patterns. FFT-associated enriched taxa such as *Prevotellaceae NK3B31 group, Tuzzerella, and Acinetobacter* showed strong positive correlations with microbial metabolites including valeric acid, caproic acid, butyric acid, and ursodeoxycholic acid. At the same time, these beneficial bacteria were negatively correlated with key liver injury markers such as ALT, AST, total bilirubin, and TNF-α, indicating a potential protective role against hepatic damage.

In contrast, taxa such as *Escherichia–Shigella, Mucispirillum,* and *Desulfovibrio* were positively correlated with elevated levels of ALT, AST, plasma endotoxin, and stool deoxycholic acid.

Overall, these findings suggest that FFT promotes the enrichment of beneficial gut microbial populations, which are associated with a favorable shift in microbial metabolite profiles (enhanced SCFAs and hydrophilic bile acids) and a concurrent reduction in liver injury biomarkers.

## DISCUSSION

Because fecal microbiota transplantation has been shown to induce infection in some patients (13), removal of bacterial cells while retaining the metabolic and protein milieu of the instillate could provide a comparable therapeutic outcome. With this hypothesis, we investigated the efficacy of fecal filtrate transplantation (FFT) in ALD for variations in hepatic function, reducing steatosis and fibrosis, restoring intestinal barrier integrity, reshaping microbial composition, and, most importantly, reprogramming metabolic pathways along the gut–liver axis.

ALD, a global health burden linked to chronic ethanol consumption, is characterized by dysbiosis, impaired intestinal barrier function, endotoxin translocation, and profound metabolic disturbances, including disrupted short-chain fatty acid (SCFA) production, bile acid imbalance, and impaired mitochondrial energy metabolism. Our results show that FFT attenuated these hallmarks by markedly reducing serum AST, ALT, and bilirubin, and fibrosis (ACTA2 and TGFβ mRNA and Col1α1 protein) markers while downregulating IL-6, TNF-α, and TLR4 and upregulating the anti-inflammatory cytokine IL-10, consistent with prior reports that microbiota-targeted therapies reduce inflammation and improve outcomes in ALD (30–36).

Mechanistically, FFT strengthened intestinal integrity by increasing ZO-1 (mRNA and protein) and Muc2, restoring intestinal IL-22 expression, upregulating the IL-22-dependent antimicrobial peptides Reg3β/γ, and reducing endotoxemia, thereby interrupting the gut–liver inflammatory loop central to ALD progression (37, 38).

While FFT did not restore microbial α-diversity, it remodeled microbial composition by depleting pathogenic taxa (*Escherichia–Shigella, Proteus, Desulfovibrio*) and enriching commensals (*Prevotellaceae NK3B31 group, Monoglobus, Acinetobacter*), highlighting that functional microbial shifts rather than diversity *per se* drive recovery (3, 39–41). Notably, abstinence showed the highest α-diversity among all groups, likely reflecting unconstrained natural recovery of the native gut community in the absence of any exogenous microbial input, whereas FFT’s defined, acellular composition drove a more targeted compositional shift without a corresponding increase in overall diversity. We also noted an increase in *Enterobacter* following FFT (Fig. 3F), a genus that includes both commensal and opportunistic pathogenic species. As 16S rRNA sequencing resolves taxa only to the genus level, this finding cannot be attributed to a specific species, and it is possible that FFT enriches non-pathogenic or facultative *Enterobacter* populations rather than pathogenic strains. FFT also produced a relative increase in *Monoglobus*, a genus specialized in dietary fiber degradation. While this link was not directly tested, this observation is consistent with the concurrent enrichment of stool SCFAs (butyric, valeric, and isobutyric acids) and the significant enrichment of the butyrate metabolism pathway in the stool metabolome, suggesting a plausible, though unconfirmed, connection between enhanced fiber-associated taxa and SCFA-driven gut–liver axis recovery.

FMT has been widely studied as a microbiota-based therapeutic, not only in recurrent *Clostridioides difficile* infection but also in metabolic disorders and liver diseases, including ALD, where it has been shown to reduce steatosis, inflammation, and gut–liver axis disruption (6, 7, 9, 10). However, concerns regarding safety and regulatory challenges associated with transferring live microbes have driven interest in FFT, which retains bioactive microbial-derived components while avoiding risks of bacterial colonization. Our study extends this concept to ALD, providing clinical evidence that FFT confers hepatoprotection, with metabolic reprogramming of the gut–liver axis emerging as a prominent associated mechanism.

Metabolomic profiling revealed a clear metabolic reprogramming: FFT-enriched hepatoprotective SCFAs such as butyrate and valerate, which are known to reinforce gut barrier integrity and dampen hepatic inflammation (20, 42, 43), while simultaneously elevating ursodeoxycholic acid and chenodeoxycholic acid and reducing toxic secondary bile acids like deoxycholic acid, thereby restoring a bile acid pool favorable for hepatoprotection (25, 44). At the hepatic level, FFT uniquely enhanced mitochondrial β-oxidation, methionine and amino acid metabolism, and cholesterol regulation by SREBF, reflecting improved energy balance, antioxidant defense, and lipid handling—all of which are impaired in ALD (28, 29, 45). These findings emphasize that the efficacy of FFT is substantially associated with metabolite-level changes, distinguishing our work from earlier FFT studies that highlighted phage- and immune-mediated effects. For example, Ott et al. demonstrated that sterile fecal filtrates resolved symptoms in recurrent *Clostridioides difficile* infection (14); Rasmussen et al. reported that fecal virome transfer from lean donors reduced weight gain and normalized glucose tolerance in obese mice (15); in ulcerative colitis, FFT induced durable immune modulation characterized by increased IL-10 and reduced IL-4 (46); and in preterm piglets, FFT prevented necrotizing enterocolitis more effectively than FMT by reducing Proteobacteria and enhancing viral diversity (47). While these studies established FFT as an effective and safe intervention, they did not directly interrogate its metabolic basis. Our work extends this knowledge by showing an association between FFT efficacy in ALD and restoration of gut–liver metabolic pathways.

This aligns with obesity models in which fecal virome transfer improved glucose tolerance and reduced weight gain, as well as metabolite-based FMT studies, such as those by Mao et al., which showed modulation of immunity and metabolism in obese mice (48), and Gedgaudas et al., demonstrated that cell-free fecal preparations transferred indole derivatives and bile acid intermediates that reduced intestinal inflammation (49). However, both studies noted that the absence of live microbial metabolism limited sustained efficacy compared to FMT. Consistently, our correlation analysis revealed that FFT-enriched taxa (*Prevotellaceae* NK3B31 group, *Acinetobacter*) were positively associated with SCFAs and ursodeoxycholic acid and negatively correlated with ALT, AST, and bilirubin, while pathogenic taxa (*Escherichia–Shigella, Desulfovibrio*) correlated with endotoxemia and hepatotoxic bile acids. Collectively, these results indicate that FFT’s therapeutic effects in ALD were closely associated with metabolic rewiring of the gut–liver axis, suggesting microbial metabolites and other acellular stool components as the candidate mediators of recovery. Although FMT produced slightly superior hepatic and intestinal recovery, the parallel improvement observed with FFT suggests that much of the therapeutic benefit of microbiota-based interventions can be achieved without the transfer of live bacteria. This highlights FFT as a safer and mechanistically distinct alternative that retains substantial efficacy while minimizing biosafety concerns associated with conventional FMT.

Nonetheless, this study has certain limitations. It was conducted in a murine model, which may not fully reflect human ALD. The intervention and follow-up period were short, leaving the durability of FFT unassessed. Although FFT clearly reprogrammed metabolism and improved liver outcomes, the specific active mediators were not explored, leaving causality indeterminate. While the filtration step in FFT preparation reduces infection risk, it also introduces practical considerations for clinical translation. Sterile filtration at scale requires standardized protocols to ensure consistent removal of bacterial cells, along with quality-control testing. These are addressable process-development considerations, similar to those already managed for other filtration-based biological preparations, and represent a practical next step toward clinical translation of FFT. Finally, other acellular components of the fecal filtrate, such as bacteriophages or host-derived molecules, require further systematic characterization to determine their contribution to the observed benefits.

### Conclusion

In conclusion, this study provides a proof of concept that FFT can effectively attenuate ALD by improving hepatic injury, restoring intestinal barrier integrity, reshaping microbial communities, and reprogramming metabolic pathways along the gut–liver axis. FFT enriched beneficial short-chain fatty acids and hepatoprotective bile acids while activating hepatic pathways involved in mitochondrial β-oxidation, methionine metabolism, and lipid handling, thereby reversing key metabolic defects of ALD. By transferring bioactive microbial-derived components without live bacteria, FFT may represent a potentially safer alternative to conventional FMT while retaining substantial therapeutic efficacy. These findings highlight FFT as a promising microbiota-based intervention for ALD, warranting further studies to identify its active mediators, confirm durability, and evaluate clinical translatability in patients.

## MATERIALS AND METHODS

### Animal experimentation and diets

Male C57BL/6 mice aged 6–8 weeks and weighing 20–25 g were obtained from the Center for Comparative Medicine (CCM), ILBS. All animals were maintained in 50%–70% humidity- and temperature-controlled rooms (21–25°C) at our institute’s specialized animal facility under specific pathogen-free conditions. During the acclimatization period, the mice were fed a semi-liquid diet for one week. Mice were then fed incremental alcohol in the Lieber-DeCarli liquid diet (Cat no. F1258SP, Bio-Serv, USA) with thioacetamide to develop the ALD model as described earlier (50). Control mice were pair-fed a non-alcoholic isocaloric Lieber-DeCarli liquid diet (Cat no. F1259SP, Bio-Serv, USA; calories equated with maltodextrin). For FFT, the donor mice were fed control liquid diet. ALD recipients were also fed control liquid diet for seven days post-FFT/abstinence. Body weight was measured every week for each animal, whereas food intake was assessed once every two days.

### Fecal microbiota/filtrate transplant

For this study, six mice were used for the donor group. ALD was established in four groups of animals (*n* = 8/group at initiation: ALD, abstinence (Abst), FMT, and FFT), accounting for anticipated mortality associated with the 12-week ethanol/thioacetamide feeding protocol. Due to mortality during model induction, *n* = 6 mice/group were available for all downstream analyses, including biochemical, histological, gene expression, 16S rRNA, and metabolomic assessments. The stool from donors was collected and immediately processed for FMT/FFT. Fresh stool (300 mg) was resuspended in 6 mL of sterile, ice-cold 0.9% saline for FMT. Stool slurry was further filtered using a 0.22-µm syringe filter to remove the bacterial cells for FFT. Recipient ALD mice received gut washes with 200 µL of PEG-4000 (Cat#81240, Sigma-Aldrich, USA). After 4 h, 200 μL of the prepared fecal filtrate slurry was administered via oral gavage. Three doses of FFT were given every other day. The experiment was terminated 1 week after the last FFT dose was given.

### Sterility assessment of fecal filtrate

To assess the removal of culturable bacteria following filtration, freshly prepared FFT was subjected to aerobic bacterial culture. Briefly, immediately after filtration through a sterile 0.22-μm syringe filter, 100 μL of freshly prepared FFT was aseptically spread onto Luria-Bertani (LB) Agar, Miller (HiMedia Laboratories Pvt. Ltd., India; Cat no. M1151) plates prepared according to the manufacturer’s instructions. As a positive control, 100 μL of the corresponding unfiltered fecal slurry was plated under identical conditions. The plates were incubated aerobically at 37°C for 48 h and examined for visible bacterial colony formation at 24 h and 48 h. The absence of visible bacterial colonies on the FFT plates, in contrast to abundant growth on the corresponding fecal slurry plates, was considered indicative of effective removal of culturable aerobic bacteria during the filtration process. The limitation that obligate anaerobic bacteria were not specifically evaluated was acknowledged.

### Tissue collection

The stool was collected pre- and post-FMT. All other tissue samples were collected at the end of the experiments. Blood was obtained via retro-orbital sinuses just before euthanasia (ketamine, 80–100 mg/Kg +xylazine, 8–10 mg/Kg). Tissue samples were weighed and divided into two groups—one set snap-frozen in liquid nitrogen and stored at −80°C till further processing. The second group for histopathology was fixed in 10% formalin.

### Liver function tests

Serum levels of alanine aminotransferase (ALT), aspartate aminotransferase (AST), bilirubin, and albumin were measured from retro-orbitally collected blood samples before starting the treatment and post day 7 of transplant using an Olympus AU5400 automatic analyzer (Olympus Optical, Tokyo, Japan).

### Histological examination

Liver lobes from all groups were collected, sectioned, and a portion was used for histological analysis. These fragments were fixed in a 10% formaldehyde solution for 24 h and then processed into paraffin blocks. Histological sections were stained with hematoxylin and eosin (H&E) to assess steatosis, and Masson’s trichrome (MT) staining to evaluate fibrosis. Immunohistochemical staining was performed to assess the extent of fibrosis using antibodies against Col1α1 (# PA5-29569, Thermo Fisher, USA) on hepatic tissue and ZO-1 (#AN300738L, Elabscience, USA) on ileal tissue to assess permeability. The sections were washed and incubated with an appropriate biotinylated secondary antibody and then with streptavidin-horseradish peroxidase complex (PolyExcel HRP/DAB Detection System Two Step Universal Kit, #PEH002, PathnSitu Biotechnologies, USA). Sections were then counterstained with hematoxylin (#S034, HiMedia). The sections were observed with the help of a light microscope (EVOSTM M5000, Thermo Fisher Scientific, USA) at a magnification of 10×, 20×, and 40×.

### Gene expression analysis by real-Time polymerase chain reaction

RNA was extracted from the liver and ileum tissues of mice using TRIzol (Cat no. 15596026, Thermo, USA) and subsequently reverse transcribed into cDNA using the RevertAid First Strand cDNA Synthesis Kit (Cat no. AB1453A, Verso cDNA kit, Thermo, USA). The gene expression of pro-inflammatory markers (IL-6, TNF-α, TGF-β, and TLR4) and the anti-inflammatory marker IL-10 was assessed in the hepatic tissues. In ileal tissues, the expression of tight junction proteins (claudin-3, zona occludens-1, and occludin), antimicrobial peptides (Reg3β and Reg3γ), IL-22, and mucin layer (MUC2) was evaluated. The ΔΔCT method was used to calculate gene expression using 18S gene expression as the control. The PCR amplification involved an initial enzyme activation step at 94°C for 3 min, followed by 35 cycles of denaturation at 94°C for 30 s, annealing at 56°C for 30 s, and elongation at 72°C for 45 s. Primer sequences used are listed in Table S1.

### Measurement of liver cytokines

Hepatic protein levels of TNF-α, IL-6, and IL-10 were measured using Mouse Uncoated ELISA Kits (Elabscience, Houston, TX) according to the manufacturer’s instructions. Briefly, liver tissue was homogenized, and the resulting supernatant was used for cytokine quantification. Plates pre-coated with capture antibody were incubated with standards or liver homogenate samples (100 μL/well) for 1 h at room temperature, followed sequentially by detection antibody and Avidin-HRP incubation, with washing steps performed between each addition. Color development was achieved using TMB substrate and terminated with sulfuric acid stop solution. Absorbance was read at 450 nm, and cytokine concentrations in each sample were calculated by interpolation from the corresponding standard curve.

### Gut microbiota analysis

Gut microbiota composition was analyzed using 16S rRNA sequencing. Fecal DNA was extracted using a SPINeasy DNA Kit (SKU: 116,547,050, MP Biomedicals) according to the manufacturer’s protocol. A 16S rRNA sequencing library was constructed according to the 16S metagenomics sequencing library preparation protocol (Illumina, USA), targeting the V3–V4 hypervariable regions of the 16S rRNA gene. All the samples were run together for sequencing on the NovaSeq 2000 sequencing platform. The reads were trimmed to remove PCR primers and adaptors to finally have a sequence length of 300 bases. Sequences with a PHRED score greater than 30 were considered for downstream analysis. Amplicon sequence variants (ASVs) were identified using the DADA2 pipeline. Rare ASVs (<0.01% counts) were removed while keeping the minimum sum of reads within all samples to more than 10,000 and with occurrence in at least 60% of samples. The SILVA database was used as the taxonomic reference for ASV classification. Statistical and ecological analysis of the meta-taxonomic data were performed as described by Volant et al. (51).

### Hepatic and stool metabolome

To determine the mechanism of FFT, we performed hepatic and stool metabolomic analyses to capture the metabolic end products of various physiological processes. 50 mg of stool and liver tissue samples were processed using 400 µL of RIPA lysis buffer (Cat no. 89901, Thermo Fisher Scientific) and sonication (30 amplitude; 10 s on/off pulses in a Helix Biosciences Sonicator, India). Stool and hepatic lysates were centrifuged at 14,000 rpm for 15 min. The supernatants were collected in separate 1.5 mL Eppendorf tubes. In 100 µL of stool lysate and plasma sample, 400 µL of ice-cold methanol was added, respectively, and kept at −20°C overnight. Samples were then centrifuged at 14,000 rpm for 30 min. The supernatant was transferred to new tubes and dried in Speed-Vac at 45°C. The dried supernatant residues were reconstituted in 95% water: 5% internal standards: 5% acetonitrile) and then subjected to reverse-phase chromatography in C18 column (Cat no. 25003102130: 3 µm × 2.1 mm × 100 mm, Thermo Scientific) using ultra-high-performance liquid chromatographic system followed by high-resolution orbitrap mass spectrometry (HRMS, Q Exactive Orbitrap Mass Spectrometer (Thermo Scientific, USA). Metabolites were annotated using Compound Discoverer 3.0 (Thermo Fisher Scientific) and filtered by MAD/median for quality control. Data normalization and scaling were performed in MetaboAnalyst 6.0 (http://metaboanalyst.ca), followed by PLS-DA, heatmap generation, and multivariate statistical analyses to identify significant metabolic features.

### Statistical analyses

GraphPad Prism version eight was used for statistical analyses. Continuous variables were compared between two groups using either the Student’s *t*-test or the Mann-Whitney test, and among more than three groups using ANOVA or the Kruskal-Wallis test, as appropriate. The categorical variables were tested using either the Pearson χ^2^ test or the Fisher exact test, as appropriate. Pearson’s correlation (r^2^  > 0.7, *P* < 0.05) was plotted using Corr plot in R (version 4.4.0). For evaluating 16S rRNA data, Bray-Curtis distances were used for the PCoA. One-way ANOVA was used for continuous variables. Differential abundance testing was performed using DESeq2 within the SHAMAN pipeline, which applies Benjamini–Hochberg false discovery rate (FDR) correction by default. All p-values reported for 16S taxa are FDR-adjusted (padj). Untargeted metabolomics data were analyzed using MetaboAnalyst 6.0 (https://www.metaboanalyst.ca/). Prior to statistical analysis, metabolite intensity data were normalized by median, log-transformed, and Pareto-scaled. Supervised multivariate analysis was performed using partial least squares-discriminant analysis (PLS-DA). Model performance and predictive ability were evaluated by 5-fold cross-validation using classification accuracy, R² (goodness-of-fit), and Q² (predictive ability), and the optimal number of latent variables was selected based on the highest cross-validated Q² value. Model robustness and potential overfitting were further assessed using 100-permutation testing based on the separation distance (between/within groups) statistic. Differential metabolites were identified using one-way ANOVA together with a fold-change threshold (>1.5), and metabolites with *P* < 0.05 were considered statistically significant. Unless otherwise specified, data are presented as mean ± SEM, and a two-tailed *P* < 0.05 was considered statistically significant.

## Data Availability

The 16S fastq data have been deposited in the DDBJ BioProject database with BioProject PRJDB42988. The raw metabolomic files can be accessed from NGDC OMIX019879 under BioProject PRJCA072498.

## SUPPLEMENTAL MATERIAL

Supplemental figures spectrum.00377-26-s0001.docx Fig. S1 to S4.

Supplemental tables spectrum.00377-26-s0002.xlsx Tables S1 to S11.

ASM does not own the copyrights to Supplemental Material that may be linked to, or accessed through, an article. The authors have granted ASM a non-exclusive, world-wide license to publish the Supplemental Material files. Please contact the corresponding author directly for reuse.
