# Supplementary material for: Fecal filtrate transplantation attenuates liver and gut injury in experimental alcohol-associated liver disease
Source: Microbiol Spectr. 2026 Sep 22;14(11):e00377-26. doi: 10.1128/spectrum.00377-26 (PMC13729205; doi:10.1128/spectrum.00377-26)
Supplement: Supplementary file 1 [file spectrum.00377-26-s0001.docx]

**Fecal filtrate transplantation attenuates liver and gut injury in experimental alcohol-associated liver disease**

Nishu Choudhary^1#^, Ashi Mittal^1#^, Anupama Kumari^1^, Kavita Yadav^1^, Jaswinder Singh Maras^1^, Shiv K Sarin^2^ and Shvetank Sharma^1*^

^1^Department of Molecular and Cellular Medicine, Institute of Liver and Biliary Sciences, New Delhi, India.

^2^Department of Hepatology, Institute of Liver and Biliary Sciences, New Delhi, India.

***# Equal contribution***

**** Correspondence***

*Dr. Shvetank Sharma, PhD*

*Additional Professor,*

*Department of Molecular and Cellular Medicine,*

*Institute of Liver and Biliary Sciences, New Delhi-110070, India*

*Tel: 011-46300000, Fax 011-26123504*

[*shvetanks@gmail.com*](mailto:shvetanks@gmail.com)

**Keywords:** Alcohol-associated liver disease, Gut microbiota, fecal-filtrate transplant, metabolome

**Supplementary Figures:**

**S. Figure 1: (A)** Aerobic bacterial culture of FFT and FMT on LB agar plates. **(B)** Body weight of animals **(C)** Liver to body weight ratio. **(D)** mRNA expression of hepatic Il6, Tnfα, Tlr4, and Il10 **(E)** Acta2 and Tgfβ. Data presented as mean ± SEM (standard error mean). *p < 0.05, **p < 0.01, ***p < 0.001 shows inter-group statistics; #p < 0.05, ##p < 0.01, ###p < 0.001 is with respect to the ALD group.

**S. Figure 2: Hepatic and intestinal recovery between FFT and FMT. (A)** Serum AST, ALT **(B)** Bilirubin and albumin levels between FFT and FMT. **(C)** Representative micrographs of liver histology by hematoxylin and eosin (H&E), Masson’s trichome (MT). **(D)** mRNA expression of ileal tight junction proteins- Zo-1, Cldn3, Ocln. **(E)** Plasma endotoxin levels. Data presented as mean ± SEM (standard error mean). *p < 0.05, **p < 0.01, ***p < 0.001 shows inter-group statistics.

**S. Figure 3: Stool metabolome: (A)** Performance assessment of the PLS-DA model using 5-fold cross-validation. Model performance is shown as classification accuracy, R², and Q² across increasing numbers of components. **(B)** Permutation testing of the PLS-DA model based on 100 permutations, with the observed statistic indicated by the red arrow (empirical p = 0.03). (**C)** Volcano plots showing differentially expressed metabolites between Abst vs ALD and **(D)**between FFT vs ALD (FC>1.5, p<0.005), upregulated and downregulated metabolites are indicated in red and blue, respectively.

**S. Figure 4: Hepatic metabolome: (A)** Performance assessment of the PLS-DA model using 5-fold cross-validation. Model performance is shown as classification accuracy, R², and Q² across increasing numbers of components. **(B)** Permutation testing of the PLS-DA model based on 100 permutations, with the observed statistic indicated by the red arrow (empirical p < 0.01). (**C)** Volcano plots showing differentially expressed metabolites between Abst vs ALD and **(D)**between FFT vs ALD (FC>1.5, p<0.005), upregulated and downregulated metabolites are indicated in red and blue, respectively.
